# The effect of rotational degree and routine activity on the risk of collapse in transtrochanteric rotational osteotomy for osteonecrosis of the femoral head—a finite element analysis

**DOI:** 10.1007/s11517-020-02137-5

**Published:** 2020-02-03

**Authors:** Pusheng Xie, Yuping Deng, Jinchuan Tan, Mian Wang, Yang Yang, Hanbin Ouyang, Wenhua Huang

**Affiliations:** 1 National Key Discipline of Human Anatomy, School of Basic Medical Sciences, Southern Medical University, 1023 ShaTai Rd, Guangzhou, 510515 People’s Republic of China; 2 Department of Anatomy, School of Basic Medicine Science, Guangdong Provincial Key laboratory of Medical Biomechanics, Southern Medical University, 1023 ShaTai Rd, Baiyun District, Guangzhou, 510515 People’s Republic of China; 3 Guangdong Engineering Research Center for Translation of Medical 3D Printing Application, Southern Medical University, 1023 ShaTai Rd, Guangzhou, 510515 People’s Republic of China; 4 Orthopaedic Center, Affiliated Hospital of Guangdong Medical University, Guangdong Medical University, Zhanjiang, 524002 People’s Republic of China

**Keywords:** Osteonecrosis of the femoral head, Transtrochanteric rotational osteotomy, Routine activity, Risk of collapse, Finite element analysis

## Abstract

To explore the mechanical mechanism and provide preoperative planning basis for transtrochanteric rotational osteotomy (TRO) procedure, a joint-preserving procedure for osteonecrosis of the femoral head. Eleven TRO finite element femurs with the most common types of necrosis were analyzed under multi-loading conditions. Thereafter, we made a comprehensive evaluation by considering the anatomy characters, daily activities, and risk indicators contain necrosis expansion trend, necrotic blood supply pressure, and the risk of fracture. The risk of fracture (ROF) is the lowest when standing on feet and increases gradually during normal walking and walking upstairs and downstairs. Compared with posterior rotation, rotating forward keeps more elements at low risk. Additionally, the correlation analysis shows it has a strong negative correlation (*R*^2^ = 0.834) with the average modulus of the roof. TRO finally decreased the stress and energy effectively. However, the stress and strain energy arise when rotated posteriorly less than 120°. The comprehensive evaluation observed that rotating forward 90°could reduce the total risks to 64%. TRO is an effective technique to prevent collapse. For the anterior and superior large necrosis, we recommend to rotate forward 60° to 90° (more efficient) or backward 180°. The methodology followed in this study could provide accurate and personalize preoperative planning.

**Graphical Abstract:**
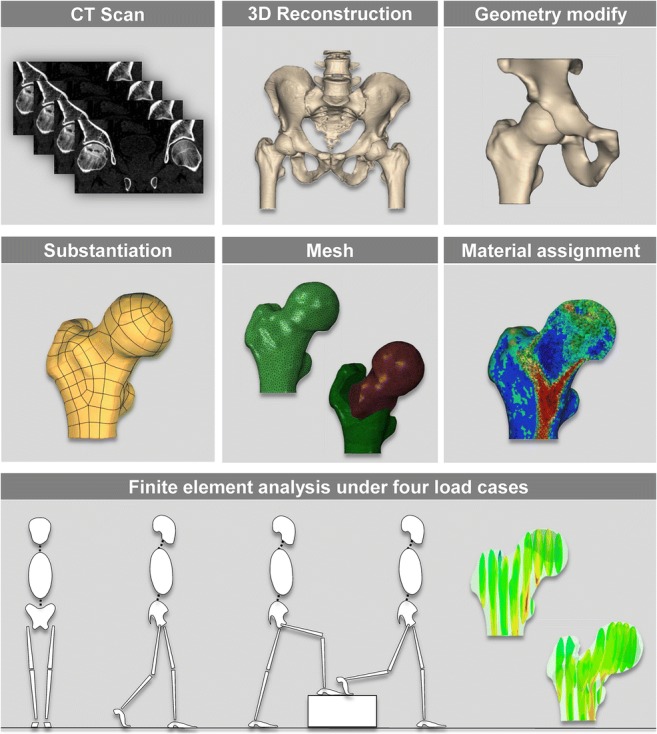
A proximal femur was reconstructed and modified using Mimics from a series of computed tomography. The models were meshed after solidified and performed different osteotomy, and then assigned material based on the Hounsfield Unit from CT images. Finally, 44 different TRO finite element femurs were analyzed under multi-loading conditions and evaluated comprehensively.

A proximal femur was reconstructed and modified using Mimics from a series of computed tomography. The models were meshed after solidified and performed different osteotomy, and then assigned material based on the Hounsfield Unit from CT images. Finally, 44 different TRO finite element femurs were analyzed under multi-loading conditions and evaluated comprehensively.

## Introduction

Osteonecrosis of the femoral head (ONFH) remains an important concern because of the increasing morbidity and high disability rate. This disease appearing as a nearly silent may lead to progressive collapse followed by degenerative arthritis of the hip [1]. ONFH occurs mainly in younger and more active patients because potentially joint replacement procedure in these circumstances is associated with high rates of failure and there has been a continued search for procedures which preserve the hip [2–4].

Transtrochanteric rotational osteotomy (TRO) has been devised as a salvage surgical treatment because it can decompress the femoral head and improve the biomechanical properties in routine activity. It is an ideal procedure moving the remained viable femoral head area to the weight-bearing locale below the acetabular roof; thus, hip joints can be preserved for long term by reducing the stresses on the necrotic zone. If there is any intact area at the anterior or posterior portion of the femoral head, then the femoral head can be rotated to move this area to the load-bearing portion.

Rotational osteotomies such as anterior rotational osteotomy posterior rotational osteotomy have been proposed. And the effect of the operation is impressive [5–7]. However, some others were unable to reproduce his results and had to give up this technically demanding procedure. Reasons for the outcome variability include improper patient selection, anatomical variation in the blood supply of the femoral head, and especially, inadequacy in preoperative plan [8–10]. The importance of rotational direction and degree has been demonstrated by some researchers [11, 12]. However, few scholars have addressed the problem of the mechanics mechanism after TRO, especially for the daily postoperative activities. In the present study, we applied finite element models with convergence analysis and mechanical experiment validation to analyze the necrosis expansion trend, necrotic blood supply pressure, and the risk of fracture by considering four routine activities after operation. The approach we have used aims to quantify the effect of rotational degree and load case on postoperative efficacy. And we tried to figure out if there were some correlation between ROF and modulus of bone. It provides theoretical basis for the selection of rotational direction and the best rotational angle.

## Materials and methods

### Geometry extraction and finite element mesh generation

**Fig. 1:**
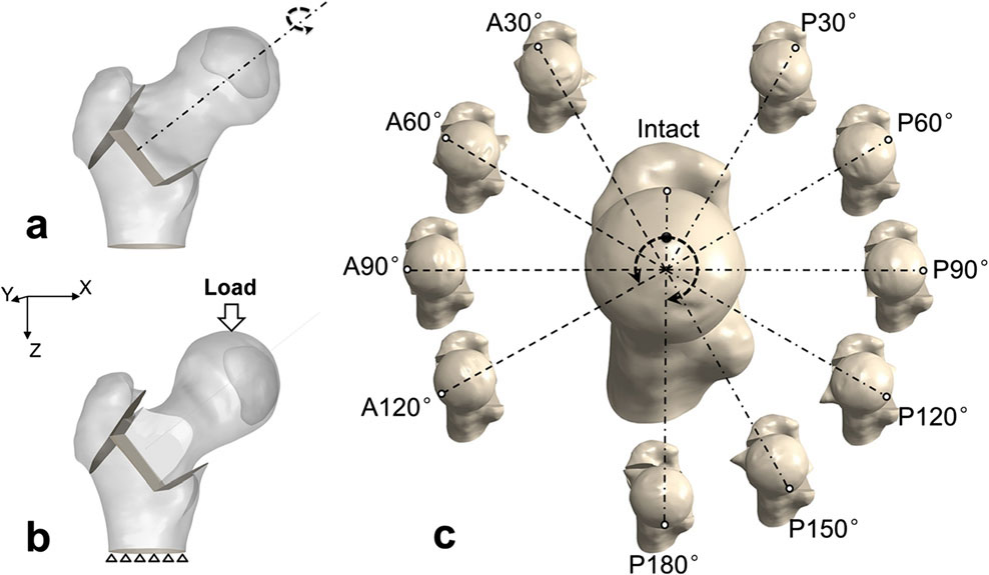
Transtrochanteric rotational osteotomy operation schematic diagram. Osteotomy was performed via three resected surface, and then the femur head was rotated through the neck axis (**a**).The boundary and loading condition were applied on the TRO femur (**b**). Different rotational degrees (**c**)

A bone structure model of the proximal femur was reconstructed using Mimics 14.0 software (Materialise, Leuven, Belgium) from a series of computed tomography images of a 34-year-old male patient with large necrosis (grade C) [13] in anterior and superior position of the femoral head. This model was imported into the Unigraphics NX 8.5 software (Siemens, Munich, Germany) to simulate TRO surgery and then 11 groups of solid model were obtained (Fig. 1). The tetrahedron elements were used to generate mesh models by Abaqus 6.14 (Dassault Systemes, Velizy-Villacoublay, France). Finally, a total of 44 different mesh models simulating 4 different load cases with an intact femur and 10 different TRO femurs were created.

### Material properties

As long as the heterogeneity of proximal femur was fully taken into account, the anisotropy of femur could be ignored. In this study, femur was modeled as an elastic and non-homogenous material by assigning specific Young’s modulus to each element by use of Mimics. In order to calculate each element’s modulus of elasticity, the Hounsfield unit (HU) was extracted from CT images and was calculated at the centroid of each element. CT intensity values were utilized in assigning mechanical properties for interior cortical and trabecular bone, modeled as a continuum of bony tissue. An intensity value for each element was calculated by averaging the brightness of pixels within the element. The bone mineral density phantom established a linear relationship between the CT scanner pixel intensity (HU) and the calcium equivalent density *ρ*. An empirical relationship specific to bone was used to convert interior bone density to elastic modulus for each element. Equations 1 and 2 were then used to find the apparent density (*ρ*, g/cm^3^) and Young’s modulus (*E*, MPa) of each element, respectively [14]. And Poisson’s ratio *v* was set to be 0.3.

1 $$ \rho =0.00069141\times \mathrm{HU}+1.026716 $$

2 $$ E=2017.3{\rho}^{2.46} $$

### Boundary and loading conditions

The distal ends of the femoral models were fully constrained as boundary condition. Four loading cases simulated the peak loading of feet standing and normal walking upstairs and downstairs were applied on the femur as recorded by Bergmann et al. [15] (Table 1). The major weight-bearing zone of the acetabulum is defined by a 40° angle around a vertical line passing through the center of the femoral head [16].

**Table 1:** Joint contact forces for the four routine activities

Load case	Magnitude(BW)	Unit vector		
		*x*	*y*	*z*
Feet standing	0.33	0	0	0.33
Normal walking	2.38	− 0.544	− 0.325	2.249
Upstairs	2.51	− 0.601	− 0.614	2.367
Downstairs	2.60	− 0.599	− 0.387	2.533

The magnitude is in units of body weight (BW)

A body weight of 600 N was assumed for the present analyses

The axis *x* is parallel to the transverse plane and pointed to the interior, *y* is toward the front, and *z* is parallel to the idealized midline of the femur and toward inferior

### Convergence test of the FEA models

The convergences of the FEA models in this study were justified by the maximum von Mises stress of the intact proximal femur. Five different models were created to perform the convergence test. A mesh was generally considered to be sufficiently refined when an increase in mesh resolution yielded roughly a 5%, or less, change in the result [17]. Based on the convergence test (Table 2), the average element size of 1.4 mm (percentage differences within 5%) was chosen as the base model. And we validated the model by comparing the maximum principal stress and displacement from previous studies [18, 19].

**Table 2:** Convergence test of the FEA models

	Case 1	Case 2	Case 3	Case 4	Case 5
Element type	C3D10				
Size (mm)	1.2	1.6	2	2.4	1.4
Element numbers	497,741	238,810	140,499	94,302	358,645
Node numbers	706,316	342,582	202,997	137,130	510,962
Max stress (MPa) in femur and changed ratio (%)	34.29 (0)	32.05 (6.53)	29.41 (14.23)	27.7 (19.22)	35.29 (0.29)
Max stress (MPa) in necrosis and changed ratio (%)	11.42 (0)	12.08 (5.779)	9.972 (12.68)	11.41 (0.088)	11.44 (0.175)

### Mechanical experiment validation

Literature verification and biomechanical experiment validation were performed in this study. Five fourth-generation composite femurs were examined with the same finite element modeling method mentioned above. And we carried out biomechanical experiments to test its axial stiffness and averaged strain value in the front area (left side of Fig. 6d) of the proximal femur with digital image correlation (DIC) method. And then the consistency analysis of axial stiffness and average strain of two groups were conducted to verify the effectiveness of our FEA method. The specific mechanical experiment verification method would be described below.

In order to reduce the error between samples and compare with related researches, we choose fourth-generation medium-sized composite bones (model number 3403) from Sawbones (Pacific Research Laboratories, Inc., Vashon Island, WA, USA) as the verification object. Each specimen was aligned under a simulated single leg stance (Fig. 6a). The force line (the line between the femoral head center and the femoral condyle center) was adjusted to the vertical direction by a 3D-printing fixture. The 5-cm distal femur was embedded and fixed by polymethyl methacrylate (PMMA). Then the anterior surfaces of the composite bones were painted with spray-paint for DIC with white background color and a black random speckle pattern (Fig. 6c) as previously reported [20]. This dyeing method does not alter the mechanical properties of the samples [21].

The axial stiffness tests were conducted by an electronic universal testing machine (ATES6010, China). The specimens were first pre-loaded with the testing machine in position to control a vertical load of up to 100 N. And then the loader was controlled to press down at a rate of 1 mm/min until the force is up to 2100 N. Loads and displacements were recorded and for at least 90 s after load removal for each repetition. Three loading repetitions were performed for each loading configuration, with the specimen being allowed to recover at least 10 min between replicates. Loads and displacements were collected by sensors. According to the load-displacement data, the slope of the linear region is calculated as the axial stiffness of the sample.

During mechanical testing, the anterior surfaces of the composite bones were recorded by a non-contact optical three-dimensional measuring system (ARAMIS 4M, GOM Corp., Germany) using DIC as previously reported [21, 22]. The two digital cameras with a frame rate of 4 Hz and pan angle of 22° to medial from anterior view created a stereo view of the surface from which the 3D surface, displacements, and surface strains were calculated using the DIC program.

According to the aforementioned method, five corresponding sawbones finite element models were constructed. After the mesh convergence analysis was performed, the same boundary and loading conditions were used for axial stiffness analysis. The force, displacement, and average strain of the region of interest were calculated and compared with the results of biomechanical experiments.

### Data analysis

The 11 groups of FEA model that applied 4 different loading conditions were investigated. For each case, the percentage change in the mechanical conditions of the treated femur in relation to the intact condition was evaluated in two different regions: (1) the roof of femoral head, (2) the necrotic area. A parameter was defined to quantify the risk of fracture (ROF) of the femoral head [23]:

$$ \mathrm{ROF}={\varepsilon}_{\mathrm{max}}/{\varepsilon}_{\mathrm{lim}} $$

where *ε*_max_ is the maximum absolute principal strain at each element of the model (either compressive or tensile) and *ε*_lim_ is the corresponding ultimate strain (ultimate tensile strain ${\varepsilon}_{\mathrm{lim}}^T=0.0073$ and ultimate compressive strain ${\varepsilon}_{\mathrm{lim}}^C=0.0104$) [24].

The maximum absolute principal strain was determined at each element for each of the intact femur. These elements were classified in four groups with the same number of elements by quartiles, this corresponds to I, II, III, and IV levels of ROF, respectively [25]. The higher level indicates higher risk of collapse. Then, the strain values of the TRO femurs were classified according to the groups/quartiles mentioned above. ROF greater than 1 means collapse happened. Thereafter, the number of elements falling into the different strain categories was quantified in the TRO femurs and compared with those of the intact femur. In addition, the average von Mises stress and all strain energy (ALLSE) were calculated to investigate the necrotic area.

### Statistical methods

All the data were collected and analyzed by SPSS Statistics v20 software (IBM, Armonk, NY, USA). The presented box plots represent median and first and third quartiles. The error bars indicate maximum and minimum values. Descriptive statistics and Pearson’s correlation analysis are adopted in the course of analysis.

## Results

### ROF

**Fig. 2:**
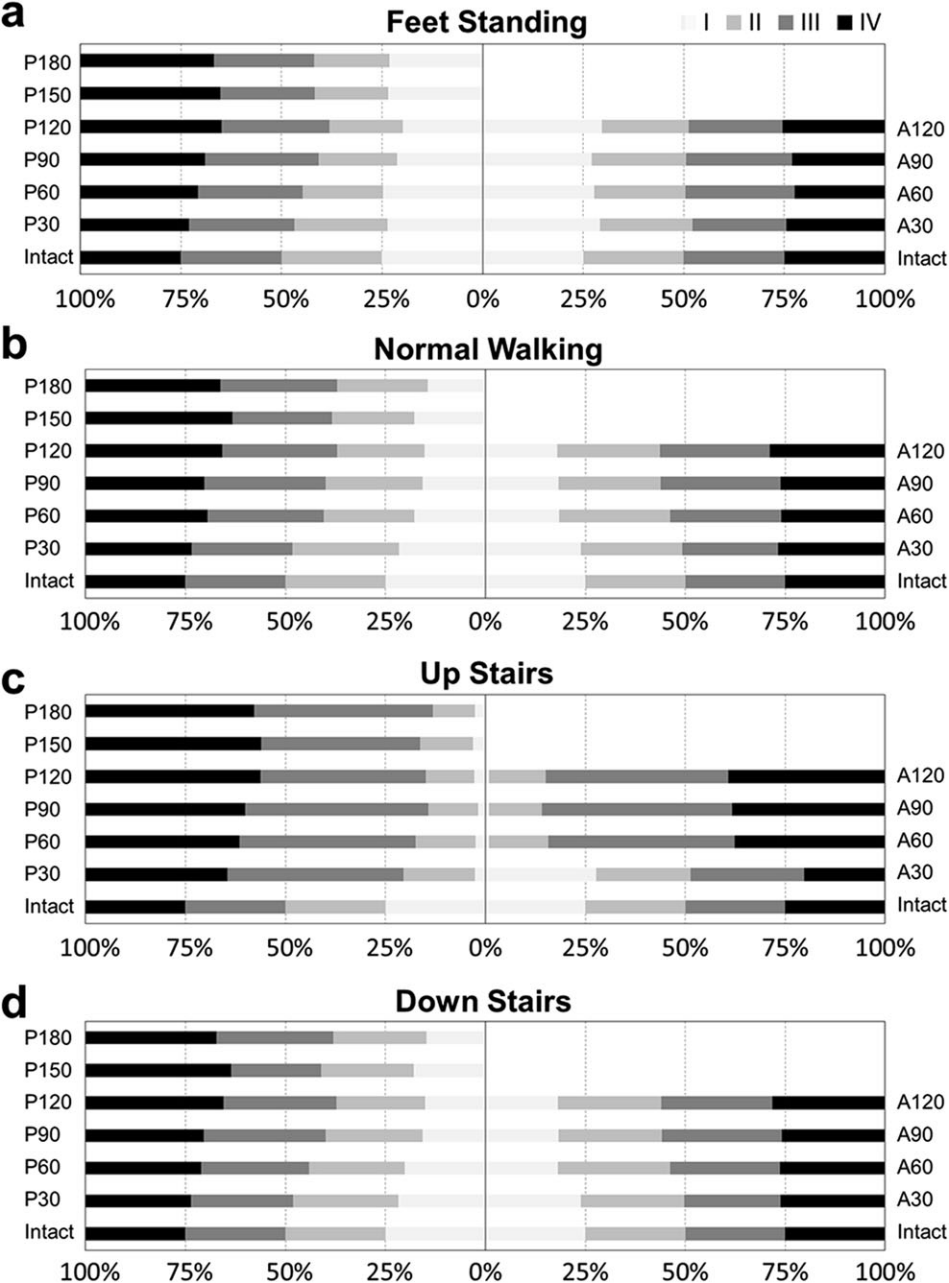
Comparison of the four levels ROF after different TRO under four load cases in relation to the intact femur

**Fig. 3:**
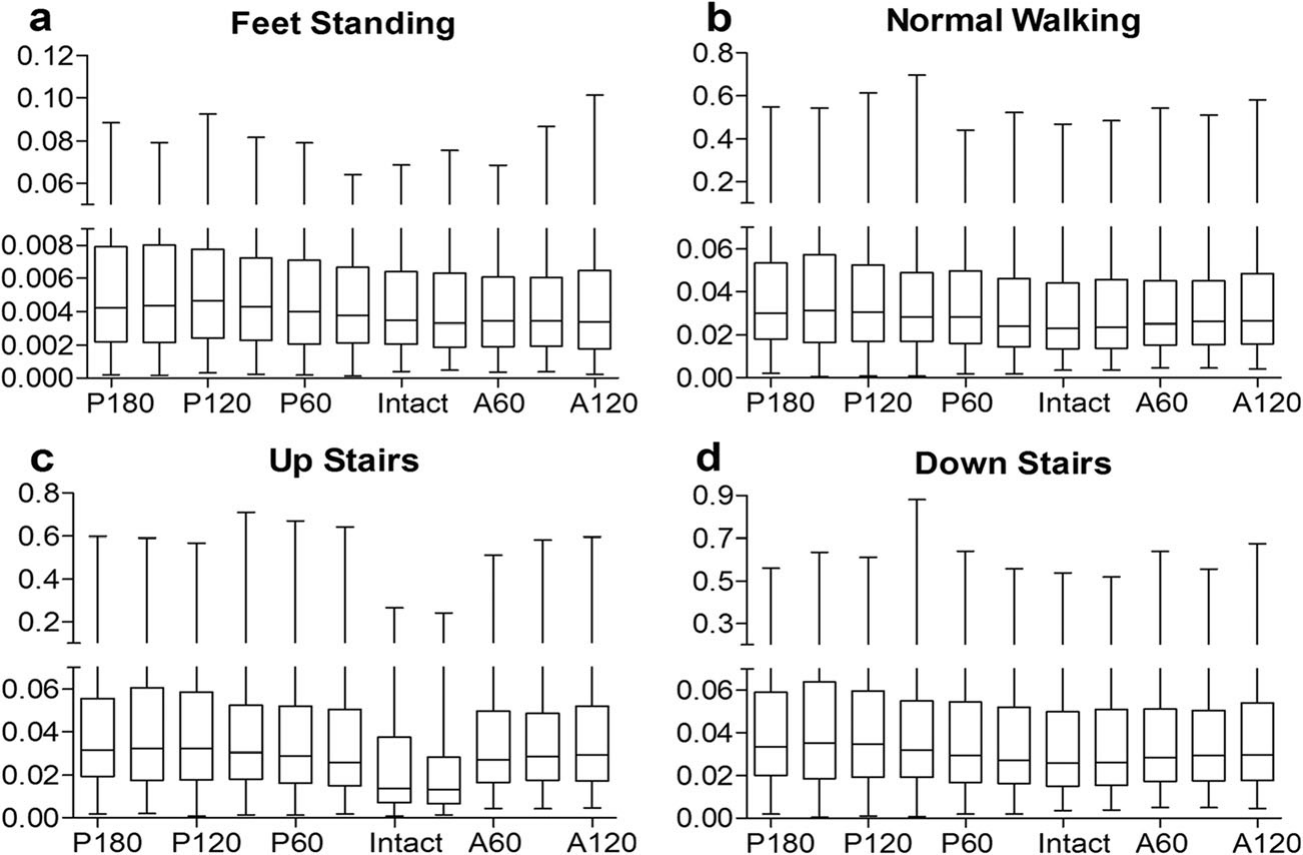
Boxplots about the ROF of the femurs after TRO under four different load cases

**Fig. 4:**
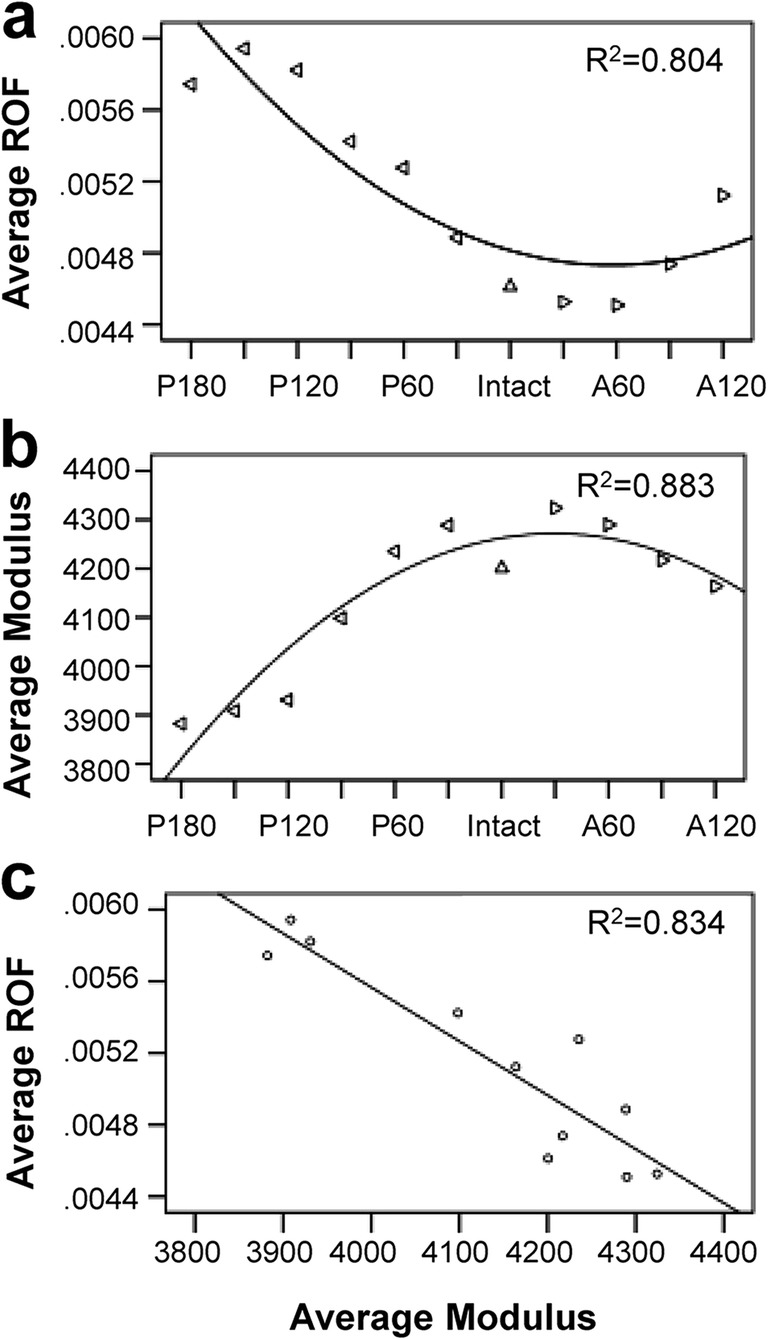
The average ROF (**a**) and modulus (**b**) of different TRO models. Correlation analysis result (**c**)

As shown in Fig. 2, the percentage of ROF of the roof area after TRO was presented in four levels. Comparing with posterior rotational osteotomy, anterior rotational osteotomy could keep more elements at a lower level of ROF. Among the four load conditions, the ROF is the lowest when standing on both feet and increases gradually during normal walking and walking upstairs and downstairs. The average and the extremal ROF graphs that reach the upper and lower bounds were given (Fig. 3). In general, the ROFs of TRO models under four load cases were all increased but less than 1 and remained at a low level after TRO. We hypothesize that it may be caused by the different property of the anterior or posterior portion of the femoral head. In order to make sure if that is related to the modulus of the roof area, we sorted out the average modulus of the roof area from all TRO models under feet standing load case. As presented in Fig. 4, it shows a strong negative correlation (*R*^2^ = 0.834). In other words, the better the bone quality at the roof of the femoral head, the lower the risk of collapse.

### Stress and ALLSE

**Fig. 5:**
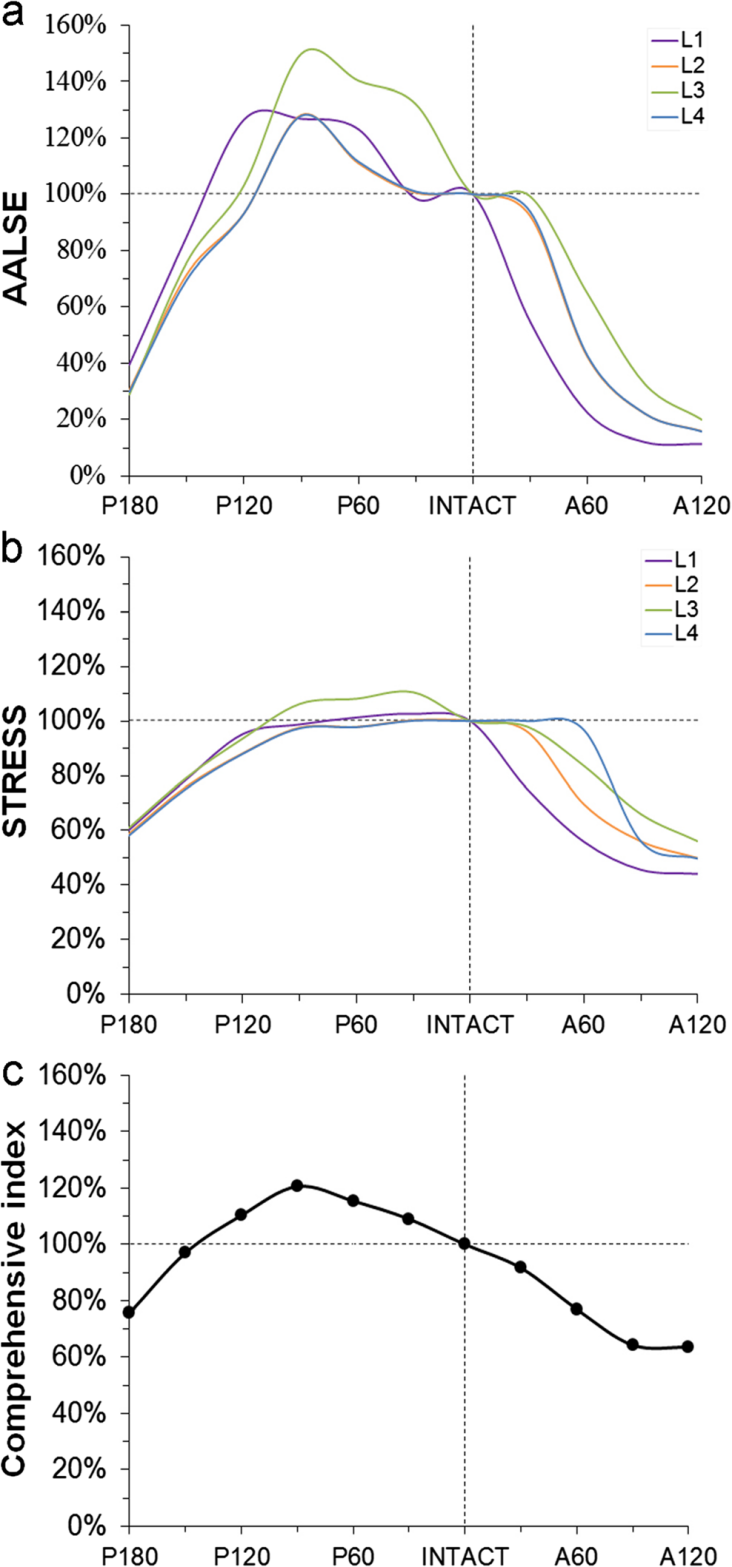
The percentage of ALLSE (**a**) and von Mises stress (**b**) remained on the necrotic area after TRO. **c** Considering four daily activities and three index values (ALLSE, stress, and ROF) comprehensively

We analyzed the ALLSE and von Mises stress under four load cases from all the elements on the necrotic region. And then the percentage of von Mises stress and ALLSE remained on the necrotic area after TRO were further calculated, as presented in Fig. 5a and b. TRO decreased the average von Mises stress and ALLSE effectively by either anterior or posterior rotation. However, the stress and strain energy arise when we rotate posteriorly less than 120°. It demonstrated that the stress reduction by the anterior rotation osteotomy was more effective as compared with that by the posterior rotational osteotomy. Finally, as shown in Fig. 5c, we made a comprehensive evaluation from the three aspects of necrosis expansion trend (ALLSE), necrotic blood supply pressure (stress), and the risk of fracture (ROF). It was observed that 90° anterior rotational could reach better results, which could comprehensively reduce the risks to 64%.

### Mechanical experiment validation

**Fig. 6:**
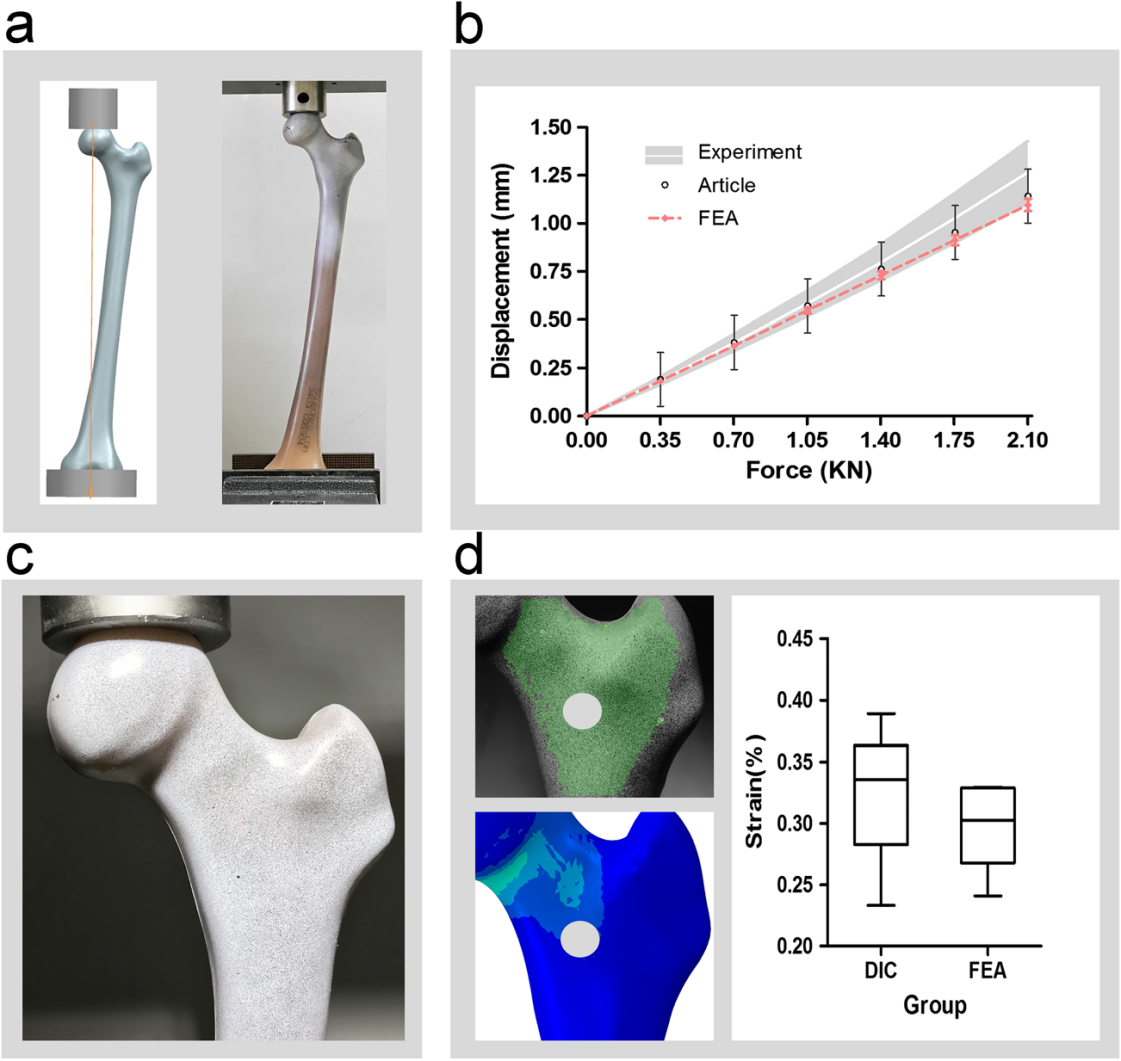
The biomechanical experimental validation. **a** The boundary and loading condition of the FEA group (left) and experimental group (right). The corresponding force and displacement data (**b**). The anterior surfaces of samples were painted with spray-paint for DIC with white background color and a black random speckle pattern (**c**). And the average strain results under 2100 N of the observation areas (left side of **d**) are compared in right side of **d**. (*n* = 5)

The biomechanical experimental validation results are presented in Fig. 6. The axial stiffness of mechanical experiment and FEA group are 1.7611 ± 0.1991 N/μm and 1.9187 ± 0.0547 N/μm, respectively. The corresponding force and displacement data are presented in Fig. 6b. It shows a strong correlation (*R*^2^ = 0.967). And the strains of the observation areas of DIC and FEA group are 0.3256% ± 0.0567% and 0.2992% ± 0.0812%, respectively. And the mutation rate is 8.12%.

## Discussion

Osteonecrosis of the femoral head is a disabling disease involving the hips of young patients. Despite many efforts, the etiology and pathogenesis of osteonecrosis still have not been identified. Therefore, treatment methods are various and are often chosen according to stage, location, and size of the necrotic area. Surgery usually involves head preserving procedures or total hip arthroplasty (THA). However, high rates of failure have been reported when performing THA in young patients, despite continuous improvement in the design and technique [3, 4]. Besides, the patients are relatively young and with high functional demands, and joint-preserving procedures should be prioritized as much as possible. As a head preserving procedure, TRO procedure itself has been widely used for the treatment of osteonecrosis of the femoral head. It provides a wide exposure and enables rotation of the healthy region to the weight-bearing area, while minimizing the risk of avascular necrosis [6, 26].

Controversy has also swirled around TRO. Fracture nonunion and subsequent collapse are the most discussed. The former is mainly related to the destruction of blood supply, while the latter is mainly related to the postoperative reconstruction of the structural mechanical environment. With a new understanding of the blood supply to the femoral head, we could perform many kinds of improved operations without destruction of blood supply [27]. In terms of mechanical reconstruction, there is lack of literature. Fewer researchers analyzed the stress distribution by FEA method and found the risk of collapse increases as the necrotic area increases. Anterior rotation and a minimum postoperative intact ratio of 34% were required to achieve successful clinical results [28].

In this study, the most common types of necrosis were analyzed by the FEA method, under multiple operating conditions (four routine activities). The convergence analysis and experiment validation were carried out for ensuring the validity of the model. In this study, two methods were used for validation. As the data of osteonecrosis model is from a living patient, it cannot be verified by direct biomechanical experiment. Therefore, we compared the stress value and distribution with related researches [11, 29], and the similar results were obtained. Meanwhile, the biomechanical experiments were promoted to verify the models from the side. The average axial stiffness of FEA model is 8.95% greater than our mechanical experiments, while 3.16% greater than 1.86 ± 0.14 N/μm of previous reports [30, 31]. Also the DIC results are very approximate. The above results indirectly prove the validity of our modeling method.

Based on effective finite element models, we made a comprehensive evaluation from the three aspects of necrosis expansion trend (ALLSE), necrotic blood supply pressure (stress), and the risk of fracture (ROF). The results show that the anterior rotational osteotomy is more efficient. Due to vascular limitation, anterior rotational osteotomy should be less than 120°. Thus, we recommend to rotate forward 60° to 90° or backward 180° by considering the anatomy characters, four daily activities and three index values comprehensively. This is consistent with the results of previous studies [11, 12]. Moreover, we found a strong negative correlation between ROF and bone quality of the roof. Average modulus of the roof area might be an effective indicator of postoperative collapse risk and help us to determine which way to rotate. Besides, we constructed finite element models by gray-scale assignment method. That is convenient for us to observe the internal stress conduction and reflect the mechanics mechanism, which is better than the previous homogenous material assignment.

Since TRO is a high technical demanding procedure, precise preoperative planning is absolutely necessary. Our study provides a good approach which would be very useful to achieve a good preoperative planning for treatment of patients with femoral head osteonecrosis. However, several limitations should be pointed out. First, since the huge amount of computation, we just analyzed the most common types of osteonecrosis under four routine activities conditions. More types should be considerate in further research. Second, the interfaces on the cutting planes were all tied without taking into account the loosening of the fixation device. Therefore, the results from the FEA might only be interpreted under the well-fixed condition. Third, the femoral FEA models were simplified as isotropy. von Mises criterion does not take into account hydrostatic pressure, and there are some limitations in judging the yield of bone. In the follow-up study, through further experimental research, it is expected to obtain more real and accurate material strength parameters of human bone. Subsequent research of yield criterion and nonlinear analysis will help to further reveal the mechanical mechanism of necrosis and collapse of femoral head.

## Conclusions

In conclusion, TRO technique can effectively reconstruct the mechanical environment of the weight-bearing area of the femoral head, reduce the stress and energy, and collapse the risk of the necrotic area. For the most necrosis located area, anterior and superior position of the femoral head, we suggest to rotate forward 60° to 90° or backward 180° by considering the anatomy characters, four daily activities and three index values comprehensively. Moreover, we firmly believe that digital medicine technology and FEA method can provide more accurate and personalized preoperative planning for patients, which could improve postoperative effect and reduce complications.
